# “Scanning mutagenesis” of the amino acid sequences flanking phosphorylation site 1 of the mitochondrial pyruvate dehydrogenase complex

**DOI:** 10.3389/fpls.2012.00153

**Published:** 2012-07-16

**Authors:** Nagib Ahsan, Kirby N. Swatek, Jingfen Zhang, Ján A. Miernyk, Dong Xu, Jay J. Thelen

**Affiliations:** ^1^Department of Biochemistry, University of MissouriColumbia, MO, USA; ^2^Interdisciplinary Plant Group, University of MissouriColumbia, MO, USA; ^3^Department of Computer Science, University of MissouriColumbia, MO, USA; ^4^Plant Genetics Research Unit, USDA, Agricultural Research Service, University of MissouriColumbia, MO, USA

**Keywords:** KiC assay, mass specrometry, mitochondrial, phosphorylation site, pyruvate dehydrogenase complex, synthetic peptides

## Abstract

The mitochondrial pyruvate dehydrogenase complex (mtPDC) is regulated by reversible seryl-phosphorylation of the E1α subunit by a dedicated, intrinsic kinase. The phospho-complex is reactivated when dephosphorylated by an intrinsic PP2C-type protein phosphatase. Both the position of the phosphorylated Ser-residue and the sequences of the flanking amino acids are highly conserved. We have used the synthetic peptide-based kinase client (KiC) assay plus recombinant pyruvate dehydrogenase E1α and E1α-kinase to perform “scanning mutagenesis” of the residues flanking the site of phosphorylation. Consistent with the results from “phylogenetic analysis” of the flanking sequences, the direct peptide-based kinase assays tolerated very few changes. Even conservative changes such as Leu, Ile, or Val for Met, or Glu for Asp, gave very marked reductions in phosphorylation. Overall the results indicate that regulation of the mtPDC by reversible phosphorylation is an extreme example of multiple, interdependent instances of co-evolution.

## Introduction

The mitochondrial pyruvate dehydrogenase complex (mtPDC) is a 10 MDa nanomachine consisting of multiple copies of three central enzymes (Mooney et al., 2002). Pyruvate dehydrogenase (E1) catalyzes the oxidative decarboxylation of pyruvate using thiamine-pyrophosphate as a cofactor, followed by the reductive-acetylation of lipoyl moieties covalently linked to dihydrolipoamide acetyltransferase (E2) which transfers the acetyl-group to CoASH, releasing acetyl-CoA as a product. Dihydrolipoyl dehydrogenase (E3) contains a FAD cofactor, and completes the reaction cycle by re-oxidizing the lipoyl groups of E2, using NAD^+^ as the electron acceptor.

The core of mtPDC is a pentagonal dodecahedron consisting of 60 E2 polypeptides arranged with icosahedral symmetry (Reed, 1974; Thelen et al., 1999; Smolle et al., 2006). The E1 and E3 enzymes are non-covalently associated with the E2 core structures. The quaternary structure of mtPDC E1 is an α2β2-heterotetramer (Fries et al., 2003). Overall, mtPDCs are very large structures with diameters of up to 50 nm. They can be easily visualized *in vitro* by either negative staining transmission electron microscopy (Stoops et al., 1992) or cryo-electron microscopy (Milne et al., 2006). The complex molecular architecture allows enhanced catalytic efficiency, achieved by a combination of active-site coupling and substrate channeling (Perham, 2000; Perham et al., 2002).

The mtPDC occupies a strategic and overlapping position in catabolic and anabolic metabolism, and activity is regulated by multiple posttranslational mechanisms including product and metabolite inhibition, and reversible multi-site phosphorylation catalyzed by intrinsic kinase (PDK) and phosphatase (PDP) components (Mooney et al., 2002; Tovar-Méndez et al., 2003). Phosphorylation of the E1α subunit by PDK inactivates the PDC, which is then reactivated by PDP-catalyzed dephosphorylation. The relative position of the Ser residue that comprises regulatory phosphorylation site 1 (Yeaman et al., 1978) plus the immediate flanking residues are conserved in all mtPDC sequences. Sites 2 and 3 have been defined only in mammalian sequences, and it is believed that *Saccharomyces cerevisiae* is phosphorylated only at site 1 (Gruhler et al., 2005; Krause-Buchholz et al., 2006).

There have been a very limited number of studies aimed at characterizing the effects of changes to amino acids flanking site 1 (Sugden et al., 1979; Mullinax et al., 1985; Hirani et al., 2011). Herein we report results from analysis of a series of systematic changes to Ser-flanking residues on phosphorylation of site 1 by PDK, using the synthetic peptide-based kinase client (KiC) assay (Huang et al., 2010).

## Methods and materials

### Synthetic peptides

A library of YHGHSMSDPGSTYR-variants consisting of 59 14-mer peptides was synthesized by the Sigma Chemical Co. (PEPscreen, St. Louis, MO, USA). The sequences are listed in Table A1. Stock solutions were prepared by dissolving the peptides in 80% (v/v) dimethylformamide in H_2_O to a final concentration of 8 mM. Peptides (Table A1) were further diluted from the stock solutions into the assays.

### Assay of PDC E1α-kinase activity

Recombinant His6-AtPDK was prepared as described previously (Tovar-Méndez et al., 2005). The synthetic peptide-based KiC assay (Huang et al., 2010) was used with minor modifications. Assays were conducted for 1 h at 37°C, with 500 rpm agitation. The assay volume was 40 μL, and peptides were tested at a final concentration of 20 μM. After assay, samples were immediately frozen and freeze-dried.

Prior to MS analysis, freeze dried-samples were dissolved by adding 40 μL of 0.1% formic acid. Half of each sample was added to a 96-well plate which was then placed onto a 10°C cooled auto-sampler. Ten microliter of each sample was analyzed using a Finnigan Surveyor liquid chromatography (LC) system coupled with an LTQ Orbitrap XL ETD mass spectrometer (Thermo Fisher, San Jose, CA). After injection, peptides were bound to a C8 Captrap (Michrom Bioresources, Auburn, CA, USA), eluted with a gradient of 1 to 30% acetonitrile (ACN) in 0.1% formic acid over a period of 15 min, and then separated using a “Magic C18” (200 Å, 5 μ bead, Michrom Bioresources) fused silica column (10 cm × 150 μ, Polymicro Technologies, Phoenix, AZ). The C8 Captrap and fused silica column were purged with two spikes of 80% ACN over a period of 6 min, before being re-equilibrated to 0.1% formic acid. Prior to MS analysis, the C18-fused silica column was pre-equilibrated with a gradient of 100–0% ACN in 0.1% formic acid to rid the matrix of any contaminating ions. Instrument settings were similar to those used previously (Huang et al., 2010). Precursor mass values were scanned with the analyzer set to FTMS: mass range, normal; resolution, 100,000; scan type, full; polarity, positive; data type, centroid; and a scan range of 300–2000 m/z. Nanospray ionization source settings were: spray voltage, 2.1 kV; capillary temp, 250°C; capillary voltage, 36 V, and tube lens, 90 V. The four most abundant ions from the precursor scan were selected for data-dependent acquisition using the ion trap-analyzer, normal mass range, normal scan rate, centroid data type, and a minimum signal threshold of 500 counts. Collision induced dissociation activation settings included: isolation width, 2.0 m/z; normalized collision energy, 35; and activation time, 30 ms. Dynamic exclusion was disabled. The multi-stage activation acquisition mode was enabled with neutral loss masses of −24.5, −32.7, −49.0, and −98.0.

### Bioinformatic analysis

The MS data were exported, processed, and compared with a decoy database consisting of the random complement of the sequences comprising the peptide library, using the SEQUEST algorithm (Proteome Discoverer 1.0, Thermo Fisher). The search parameter settings have been previously described (Huang et al., 2010). Identifications were evaluated using the XCorr versus charge state function of Proteome Discoverer, and peptide identities with a score of >13.01 (a probability of 0.05) were accepted. The XCorr values for each charge state were set to default, and no decoy hits were allowed. Spectra that passed this filtering were exported to PhosSite (in-house software, available for download at digbio.missouri.edu/p3db/tools) to assign specific sites of phosphorylation. For final validation, each spectrum was inspected manually, and accepted only when the phospho-peptide had the highest XCorr value, site-determining fragment ions allowed unambiguous localization of the phosphorylation site, and all major ions in the spectrum were accounted for.

**Figure A1:**
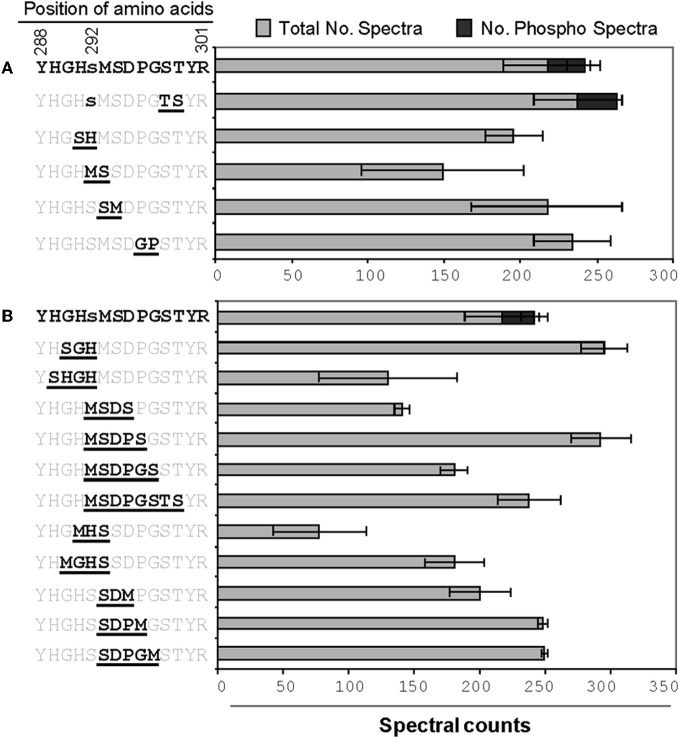
**The effects of changes to the amino acids flanking phosphorylation site 1 of *A. thaliana* PDH E1α. (A,B)** indicate double and multiple changes, respectively. Bold underlined letters in the amino acid sequence indicates the sites of change. A lower case letter indicates a potential phosphorylation site. The spectral count data presented are means of three technical replicates each for both phosphorylated and non-phosphorylated peptides.

**Figure A2:**
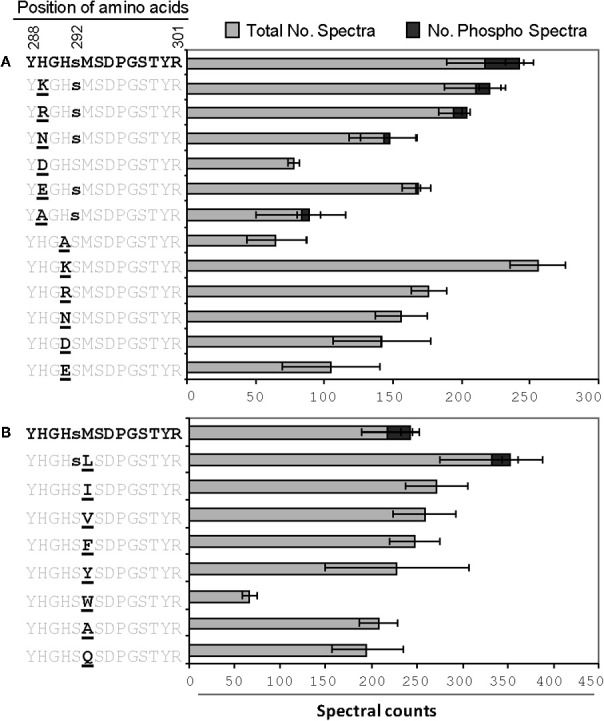
**The effects of single changes to **(A)** His_289_/His_291_, or **(B)** Met_293_.** The spectral count data presented are means of three technical replicates each for both phosphorylated and non-phosphorylated peptides.

**Figure A3:**
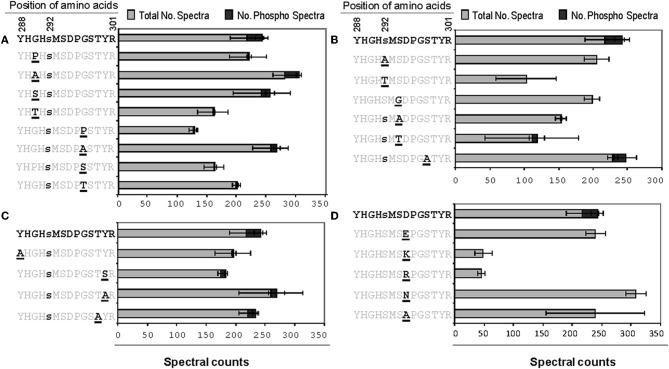
**The effects of single changes to the amino acid residues flanking phosphorylation site 1 of *A. thaliana* PDH E1α.** The spectral count data presented are means of three technical replicates each for both phosphorylated and non-phosphorylated peptides.

Data presented as heat-maps (Figures 2 and 3) are from spectral counting, and are means of three technical replicates. While we considered the possibility unlikely, it is conceivable that non-phosphorylated peptides would not ionize as well as those that are phosphorylated, that they might not “fly” as well, or might in some other way be difficult to compare. To allay any concerns, all the data for detection of both phosphorylated and non-phosphorylated peptides is presented in the Figures A1–A3.

### 3D modeling of native *A. thaliana* PDC E1α and the phosphorylation site-flanking variants

MUFOLD is an efficient and accurate protein tertiary structure prediction platform that applies whole and partial template information along with computational techniques (Zhang et al., 2010). For the target, AtPDC E1α, MUFOLD was used first to predict the secondary structure and solvent accessibility and then to scan the PDB for target/template alignments. The best templates are 1NI4_A, 2OZL_A, and 3EXE_A from the 1.2.4.1 enzyme family and 1UMD_A and 2BFF_A from the 1.2.4.4 enzyme family. These templates cover almost 95% of the target residues. However, the first 32 N-terminal target residues are either not covered by any template or the predicted secondary structures (solvent accessibilities) are not matched with the templates. A similar situation exists for some C-terminal target residues. Thus, MUFOLD was used to search and evaluate fragmental structures which fit the target termini in terms of sequence, secondary structure, solvent accessibility, and 3D structure. Finally, MUFOLD used the structural constraints from templates and fragments to build the target model. In order to predict the local structure changes that resulted from each flanking region residue substitution, e.g., M293L (peptide 11), the overall process was repeated to build a 3D model.

## Results and discussion

The E1 component of Type II PDC is an α2β2 heterotetramer. Mammalian mtPDC is regulated by multi-site phosphorylation of the E1α subunit, catalyzed by the intrinsic PDK. While site 1 is preferentially phosphorylated by PDK, saturation phosphorylation of any one of the three sites on any single E1α subunit completely inhibits PDC activity (Sugden and Randle, 1978). The PDK is docked on the inner-lipoyl domain of the dilipoyl-E2 subunits, and the flexibility of this domain allows a single kinase to phosphorylate more than one E1α subunit (Roche et al., 2003). It is believed that phosphorylation at Ser_292_ prevents thiamine-PP-induced ordering of the sequence loop carrying the phosphorylation site. The disordering of this phosphorylation-loop is caused by a steric clash between the phosphoryl-group at site 1 and the nearby Ser_294_, which nullifies a hydrogen-bonding network essential for maintaining the loop conformation (Kato et al., 2008). The disordered phosphorylation loop impedes binding of lipoyl-domains of the PDC E2 core to E1, preventing the reductive acetylation step. This results in disruption of substrate channeling, and leads to inactivation.

The first site 1 flanking sequence was experimentally determined by manual Edman sequencing of a tryptic peptide derived from bovine PDC previously incubated with γ−^32^P-ATP (Yeaman et al., 1978; Figures 4A,B). We have chosen to refer to site 1 as “Ser_292_,” based upon the sequence of *Arabidopsis thaliana* E1α (Luethy et al., 1995). During the genome-sequencing era it has been established that the position of Ser_292_ and the sequences of the flanking amino acids are highly conserved in mtPDC E1α sequences. When directly studied, it has been verified that Ser_292_ was phosphorylated by the intrinsic PDK (e.g., Bykova et al., 2003; Hirani et al., 2011).

**Figure 1:**
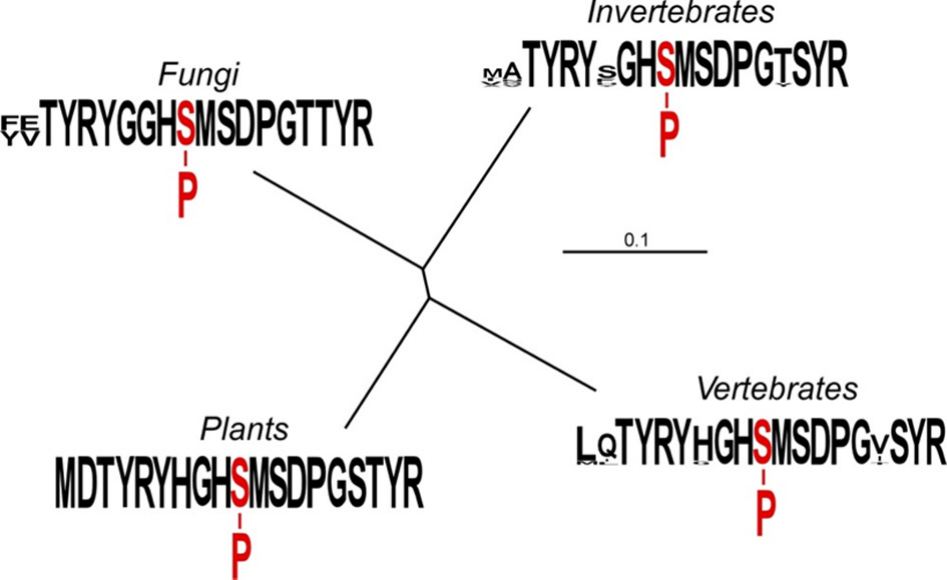
**Relationships among the amino acid sequences immediately flanking phosphorylation site 1 of mitochondrial PDC E1α.** The un-rooted tree was generated with TreeView version 1.6.6 (http://taxonomy.zoology.gla.ac.uk/rod/treeview.html). Each clade comprises 10 randomly selected sequences. The plant clade includes sequences of the “tryptic peptide” from *Pisum sativum, A. thaliana, Glycine max, Lycopersicon esculentum, Solanum tuberosum, Beta vulgaris, Medicago truncatula, Zea mays, Oryza sativa*, and *Triticum aestivum*. The sequences are identical. The vertebrate clade includes sequences from *Homo sapiens, Rattus norvegicus, Gallus gallus, Macaca fascicularis, Xenopus laevis, Danio rerio, Oryctolagus cuniculus, Ailuropoda melanoleuca, Heterocephalus glaber*, and *Capsaspora owczarzaki*. The invertebrate clade includes sequences from both nematodes and insects; *Ascaris suum, Caenorhabditis elegans, Bombyx mori, Aedes aegypti, Drosophila melanogaster, Daphnia pulex, Tribolium castaneum, Solenopsis invicta, Amblyomma maculatum*, and *Batrachochytrium dendrobatidis*. The fungal clade includes sequences from *Saccharomyces cerevisiae, Schizosaccharomyces pombe, Kluyveromyces lactis, Naumovozyma dairenensis, Clavispora lusitaniae, Meyerozyma guilliermondii, Schizophyllum commune, Fusarium oxysporum, Verticillium alboatrum*, and *Ajellomyces capsulatus*. The clade sequence presentations were assembled using WebLogo (Crooks et al., 2004).

Based upon our phylogenic comparisons, the sequences Thr_285_ to Tyr_288_, Gly_290_ to Gly_297_, and Tyr_300_ to Arg_301_ are invariant, suggesting functional necessity in terms of E1 catalysis, PDK docking or orientation, or possibly some aspect of phospho-transfer. Residue 283 is typically large and hydrophobic, although Thr or Ala is present in some invertebrate sequences (Figure 1). Residue 284 appears to be unconstrained (Asp, Glu, Val, Ile, Leu, Gln, Asn, or Ala). Residue 289 is most often Gly or His, but can also be Ser, Thr, or Phe in the invertebrate sequences. Residue 298 is most often Ser or Thr, but can also be Val, Ile, or Tyr. Residue 299 is Ser in both vertebrate and invertebrate sequences, but Thr in plants and fungi. The Ser_299_ residue is regulatory phosphorylation site 2 in mammalian PDC (Davis et al., 1977; Sugden et al., 1979; Mullinax et al., 1985).

**Figure 2:**
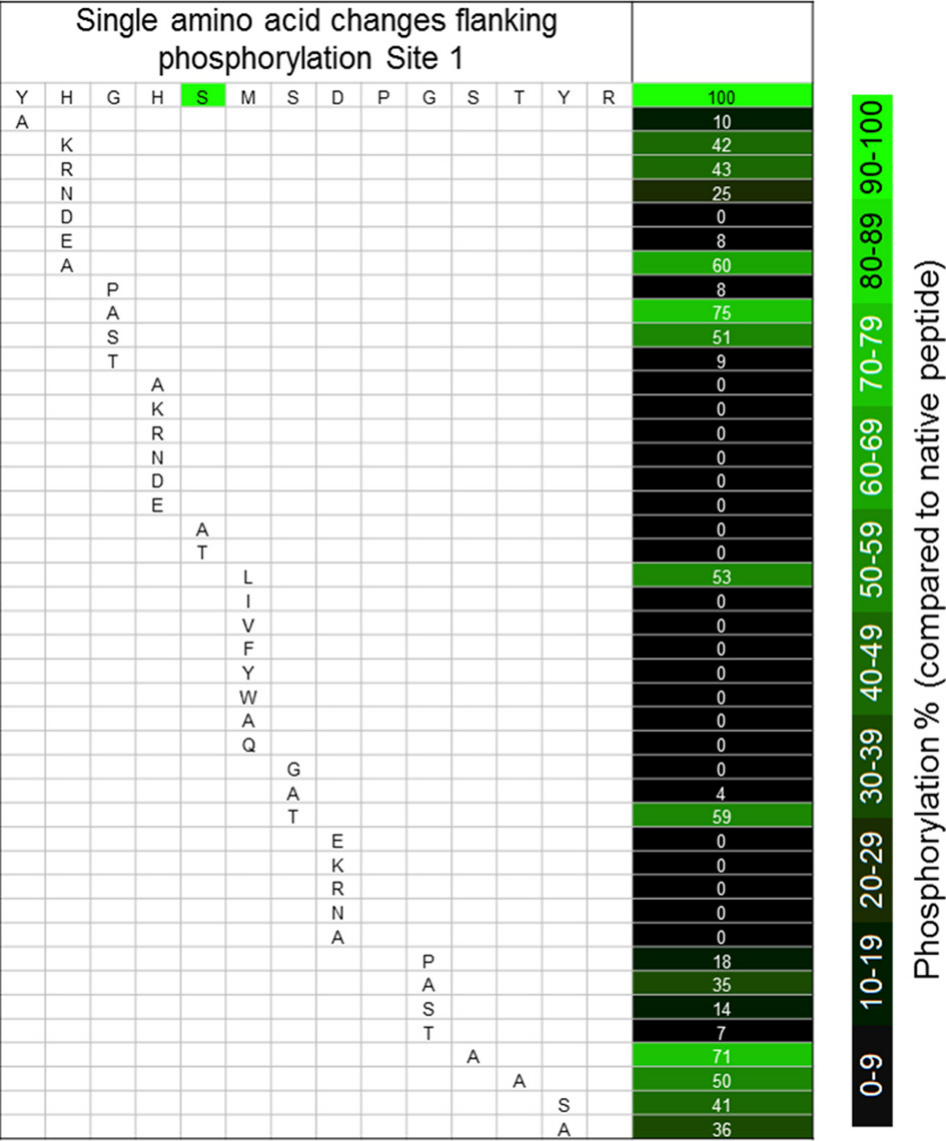
**The effects of single amino acid changes to the sequences flanking phosphorylation site 1 of *A*. *thaliana* PDC E1α.** Values are percentage changes compared with the native sequences, and are the means of three technical replicates.

Our “scanning mutagenesis” strategy, using synthetic peptides, recombinant PDK, and the quantitative KiC assay, yielded a set of results that would have been at least difficult to obtain using site-directed mutagenesis of the intact protein sequence because of the occurrence of competing effects upon catalytic activity (e.g., Hirani et al., 2011). As suggested by the results from comparative sequence analyses, the site 1 flanking sequences tolerate little change. Only a few conserved “mutations” at specific positions allowed fractional activity (Figure 2, and Huang et al., 2010). In no instance did any peptide yield a hyperactive result. While the Y288A (peptide 58) is a drastic change, it was unexpected that a change three residues distal to S_292_ would have such a large effect upon phosphorylation (Figure 2). Especially in light of the observation that the H289A (peptide 54) change had a much smaller inhibition. “Conservative” substitutions for His_289_ (Lys, Arg, Asn; peptides 36–38, respectively) all resulted in a 55% inhibition of phosphorylation of Ser_292_ (Figure 2). Changes of Gly_290_ to Ala (peptide 33) or Ser (peptide 34) inhibited phosphorylation of Ser_292_ much less than did changes to Pro (peptide 32) or Thr (peptide 35). Most changes to His_291_ (peptides 3–5, 17, 18, 27–31) completely inhibited phosphorylation, and the S292A (peptide 48) and S292T (59) peptides were not phosphorylated (Figure 2). It has previously been reported that S292T peptides were not phosphoryl acceptors (Miernyk and Randall, 1989; Huang et al., 2010).

We have chosen to present our peptide phosphorylation data as spectral counts, a relatively simple format that is being increasingly used in proteomic analyses (Mueller et al., 2008; Huang et al., 2010; Thelen and Miernyk, 2012). While our numbers are not large, they are reproducible (Figures A1–A3), and it is clear that there are not any problems detecting and quantifying either phosphorylated or non-phosphorylated peptides.

Phosphorylation of the M293L (11) peptide was inhibited 50%, but any other changes, conservative or otherwise, completely prevented phosphorylation of Ser_292_ (peptides 12–22; Figure 2, and Huang et al., 2010). The structural bases for the differences must be subtle because there do not appear to be any major perturbations (Figures 4D,E). It was previously reported that oxidation of Met_293_ to MetSO almost completely blocked phosphorylation of Ser_292_ (Miernyk et al., 2009; Huang et al., 2010), and it was proposed that this reversible change might be of regulatory importance.

The S294T substitution (peptide 60) decreased phosphorylation of Ser_292_ 50%, while changes to Ala (peptide 49) or Gly (peptide 46) inhibited it completely (Figure 2, and Huang et al., 2010). It has been proposed that Ser_294_ might be involved in thiamin-PP binding (Kato et al., 2008). Our results suggest that it is the hydroxyl-group that is necessary for phosphorylation rather than simply spacing due to amino acid size (S/T v. A).

We observed that any changes to Asp_295_, including the conservative D295E (peptide 23) change, resulted in complete inhibition of Ser_292_ phosphorylation (Figure 2). From the predicted structures, it can be seen that the D295K (Figure 4F) and D295E (Figure 4G) changes cause significant changes in the positioning/presentation of Ser_294_, Met_293_, and Ser_292_. Previous studies of the D295N change using peptide substrates with the mammalian PDK showed substantially reduced phosphorylation of S_292_, and it was proposed that Asp_295_ is involved with PDK binding (Mullinax et al., 1985). The results of Mullinax et al. (1985) were recently verified by Hirani et al. (2011) using recombinant *A. thaliana* PDC-E1 and PDK. The D295N, D295A, and D295L site-directed mutants all had greatly reduced phosphorylation of E1α.

**Figure 3:**
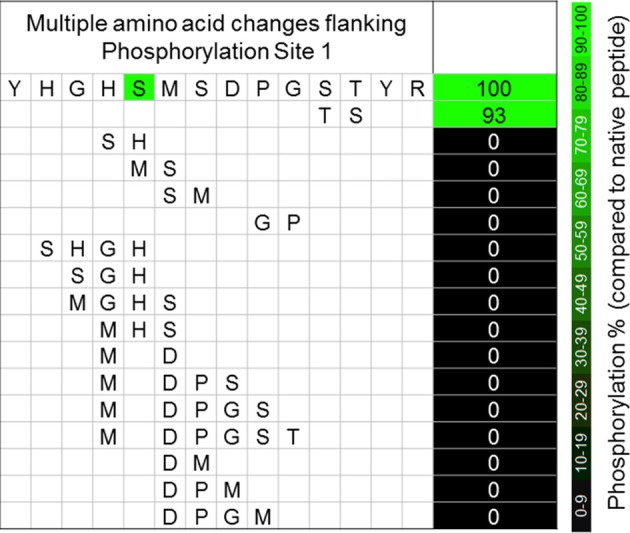
**The effects of multiple simultaneous amino acid changes to the sequences flanking phosphorylation site 1 of *A. thaliana* PDC E1α.** Values are percentage changes compared with the native sequences, and are the means of three technical replicates.

Because of an unfortunate oversight in our experimental design, we failed to systematically evaluate changes to Pro_296_. We believe, however, that the absolute conservation of Pro at this position in all E1α sequences (Figure 1), and the complete loss of phosphorylation resulting from the exchange of Pro_296_Gly_297_ for Gly_296_Pro_297_ (Figure 3) support our contention that any change from Pro at position 296 will also result in the loss of phosphorylation.

When residue 297 was changed from Gly to Pro (peptide 41), Ala (peptide 43), Ser (peptide 44), or Thr (peptide 45), phosphorylation of Ser_292_ was reduced from 65 to 90% (Figure 2). Hirani et al. (2011) reported that with the G297S and G297D site-directed mutants the phosphorylation of Ser_292_ was only inhibited approximately 30%. The phylogenetic sequence alignments revealed an absolute conservation of Gly at position 297 (Figure 1). As was found with substitutions for the N-terminal Tyr_288_, changes to the distal C-terminal Ser_298_, Thr_299_, Tyr_300_, and Arg_301_ residues significantly inhibited phosphorylation of Ser_292_ (30–60%).

**Figure 4:**
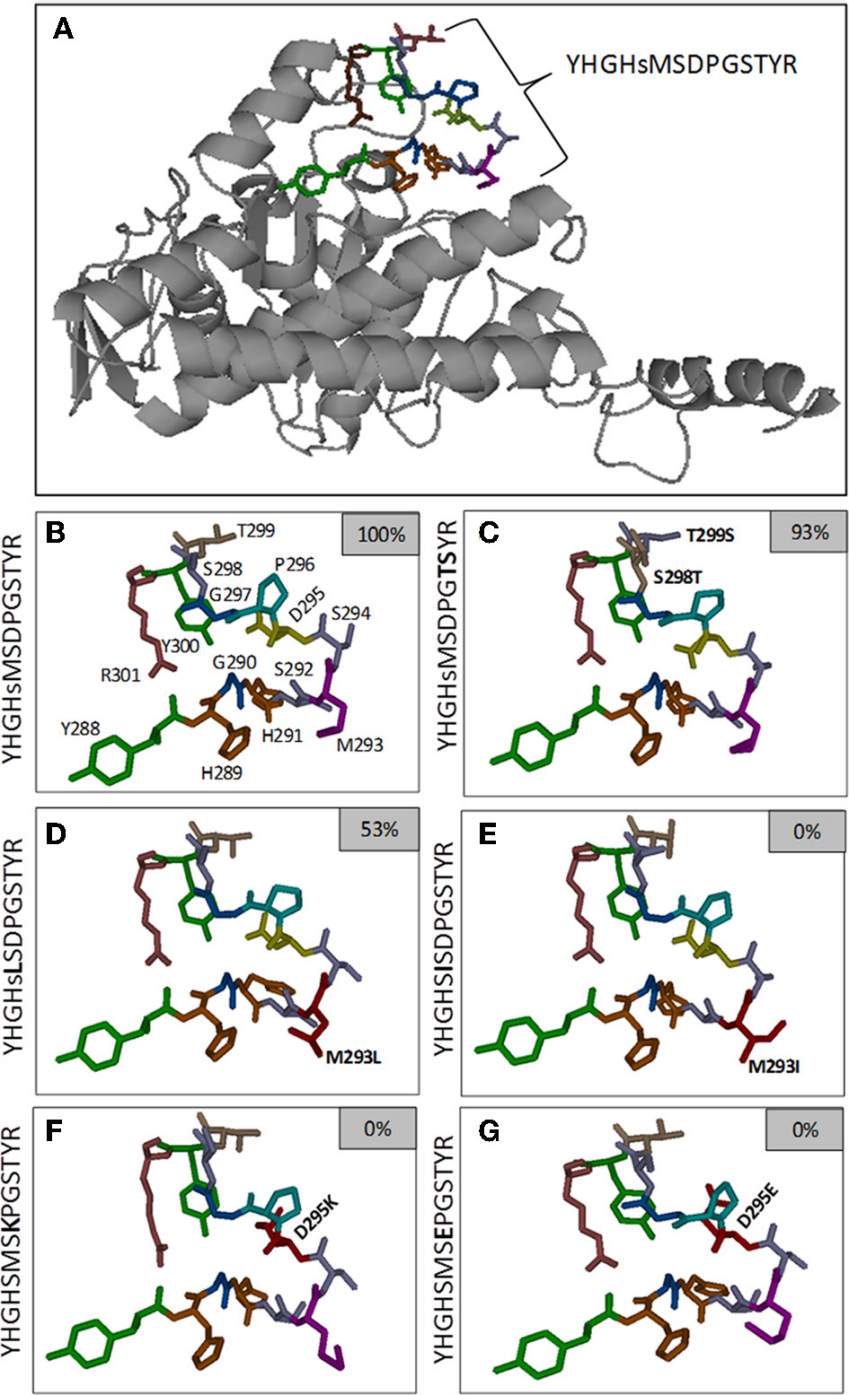
**Predicted local structure of the *A. thaliana* PDC E1α subunit, with emphasis on the phosphorylation site 1 (Ser_292_)-flanking residues.** Panel **(A)** includes the 3D representation of the full-length E1α subunit. Regulatory phosphorylation site 1 is shown as a colored stick-diagram. In panel **(B)**, an enlarged version of the sequence flanking site 1 is presented. In panels **(C–G)** the stick-diagrams of selected amino acid substitution variants are presented. The small inset boxes indicate the extent of phosphorylation relative to the native sequence.

While even a single conservative change to a flanking amino acid might substantially reduce phosphorylation of Ser_292_ (Figure 2), nearly all multiple-changes completely abolished phosphorylation (Figure 3 and Table A1). This included the simple exchanging or scrambling of neighboring residues. The multiple changes probably reflect a combination of reduced accessibility of Ser_292_ to PDK plus impaired recognition and/or binding. The single exception to this among the peptides screened was the switching of Ser_298_/Thr_299_ (peptide 2) which was without substantial effect on phosphorylation of Ser_292_ (Figure 3). This exchange converts the plant flanking-sequence to that of mammalian E1α and recreates mammalian site 2 (Figure 1). Despite this, Ser_299_ was not phosphorylated in our assays. When the predicted structure of peptide 2 (Table A1), which contains the Ser_298_/Thr_299_ to Thr_298_/Ser_299_ exchange, is examined (Figure 4C) it can be seen that in addition to other minor changes there is a significant change in the presentation of D_295_. While this does not affect phosphorylation of Ser_292_, it might contribute to Ser_299_ not being phosphorylated. It is additionally noteworthy that all previous reports of phosphorylation of mammalian site 2 have been done with either intact PDC or the E2-containing subcomplex present in the assays along with E1 and PDK.

## Conclusions

Understanding the structure-function relationship between protein kinases and their clients is integral to understanding kinase specificity. Herein we demonstrate a facile approach for comprehensively and quantitatively assessing kinase specificity, using the MS-based KiC assay. There are two potential concerns with the use of MS for studying peptide-protein interactions; accurate discrimination of isobaric peptides, and differential ionization efficiency. To control for these two problems, respectively, we performed all peptide assays independently, and expressed phosphorylation efficiency as a ratio to the non-phosphorylated species. This assumes that phosphorylated peptides ionize with similar efficiency, which might otherwise be a concern in a more dramatic mutagenic schema.

The primary sequence drives formation of the secondary structure that underlies catalysis. In the case of the peptides being studied, there are primary sequence constraints relative to binding of both the substrate pyruvate and the co-factor thiamin-PP. At the same time, the evolutionary acquisition of reversible phosphorylation as a regulatory mechanism must have included the selection of primary sequence requirements necessary for kinase (and phosphatase) recognition and binding. It is the combination of these two interacting selective considerations, along with those related to the catalytic function of E1, that likely comprise the basis for the stringent primary-sequence specificity including and flanking “Ser_292_.”

### Conflict of interest statement

The authors declare that the research was conducted in the absence of any commercial or financial relationships that could be construed as a potential conflict of interest.

## Appendix

**Table A1:** **Synthetic peptide-variants of the plant pyruvate dehydrogenase E1α phosphorylation site 1-flanking sequences**.

**Number**	**Sequence**	**MW**
1	YHGHSMSDPGSTYR	1594.6703
2	YHGHSMSDPGTSYR	1594.6703
3	YHGSHMSDPGSTYR	1594.6703
4	YHSGHMSDPGSTYR	1594.6703
5	YSHGHMSDPGSTYR	1594.6703
6	YHGHMSSDPGSTYR	1594.6703
7	YHGHMSDSPGSTYR	1594.6703
8	YHGHMSDPSGSTYR	1594.6703
9	YHGHMSDPGSSTYR	1594.6703
10	YHGHMSDPGSTSYR	1594.6703
11	YHGHSLSDPGSTYR	1576.7139
12	YHGHSISDPGSTYR	1576.7139
13	YHGHSVSDPGSTYR	1562.6982
14	YHGHSFSDPGSTYR	1610.6982
15	YHGHSYSDPGSTYR	1626.6931
16	YHGHSWSDPGSTYR	1649.7091
17	YHGMHSSDPGSTYR	1594.6703
18	YHMGHSSDPGSTYR	1594.6703
19	YHGHSSMDPGSTYR	1594.6703
20	YHGHSSDMPGSTYR	1594.6703
21	YHGHSSDPMGSTYR	1594.6703
22	YHGHSSDPGMSTYR	1594.6703
23	YHGHSMSEPGSTYR	1608.6859
24	YHGHSMSKPGSTYR	1607.7383
25	YHGHSMSRPGSTYR	1635.7445
26	YHGHSMSNPGSTYR	1593.6863
27	YHGKSMSDPGSTYR	1585.7063
28	YHGRSMSDPGSTYR	1613.7125
29	YHGNSMSDPGSTYR	1571.6543
30	YHGDSMSDPGSTYR	1572.6383
31	YHGESMSDPGSTYR	1586.6540
32	YHPHSMSDPGSTYR	1634.7016
33	YHAHSMSDPGSTYR	1608.6859
34	YHSHSMSDPGSTYR	1624.6809
35	YHTHSMSDPGSTYR	1638.6965
36	YKGHSMSDPGSTYR	1585.7063
37	YRGHSMSDPGSTYR	1613.7125
38	YNGHSMSDPGSTYR	1571.6543
39	YDGHSMSDPGSTYR	1572.6383
40	YEGHSMSDPGSTYR	1586.6540
41	YHGHSMSDGPSTYR	1594.6703
42	YHGHSMSDPPSTYR	1634.7016
43	YHGHSMSDPASTYR	1608.6859
44	YHGHSMSDPSSTYR	1624.6809
45	YHGHSMSDPTSTYR	1638.6965
46	YHGHSMGDPGSTYR	1564.6597
47	YHGHSMSDPGSTSR	1518.6390
48	YHGHAMSDPGSTYR	1578.6754
49	YHGHSMADPGSTYR	1578.6754
50	YHGHSASDPGSTYR	1534.6669
51	YHGHSQSDPGSTYR	1591.6884
52	YHGHSMSAPGSTYR	1550.6805
53	YHGHSMSDPGATYR	1578.6754
54	YAGHSMSDPGSTYR	1528.6485
55	YHGASMSDPGSTYR	1528.6485
56	YHGHSMSDPGSTAR	1502.6441
57	YHGHSMSDPGSAYR	1564.6597
58	AHGHSMSDPGSTYR	1502.6441
59	YHGHTMSDPGSTYR	1608.6859
60	YHGHSMTDPGSTYR	1608.6859
